# Psychometric Validation of the STEM Disciplines Attitude Scale (SDAS): Investigating Measurement Invariance and Psychological Correlates in Italian High School Students

**DOI:** 10.3390/ejihpe16090125

**Published:** 2026-08-26

**Authors:** Angelo Fumia, Luana Sorrenti, Carmelo Mario Vicario, Francesca Ferraioli, Simona Massimino, Rosa Angela Fabio, Pina Filippello, Daniele Mollaioli

**Affiliations:** 1 Department of Health Sciences, ‘Magna Graecia’ University of Catanzaro, 88100 Catanzaro, Italy; 2 Department of Clinical and Experimental Medicine, University of Messina, 98125 Messina, Italy; luana.sorrenti@unime.it (L.S.); gfilippello@unime.it (P.F.); daniele.mollaioli@unime.it (D.M.); 3 Department of Cognitive, Psychological, Educational and Cultural Studies, University of Messina, 98121 Messina, Italy; carmelomario.vicario@unime.it (C.M.V.); francesca.ferraioli@unime.it (F.F.); simona.massimino@unime.it (S.M.); 4 Department of Biomedical and Dental Sciences and Morphofunctional Imaging, University of Messina, 98125 Messina, Italy; rafabio@unime.it

**Keywords:** STEM interest, secondary education, hard sciences, psychometric validation

## Abstract

Promoting positive student attitudes toward STEM is essential for fostering innovation; however, many existing measures do not adequately distinguish attitudes across specific disciplines, particularly within hard sciences such as physics and chemistry. This study presents the development and validation of the STEM Disciplines Attitude Scale (SDAS). Across three studies involving 2577 high school students, Exploratory and Confirmatory Factor Analyses supported a 25-item, five-factor structure assessing attitudes toward Mathematics, Physics, Chemistry, Engineering, and Science and Technology. The SDAS showed good internal consistency and strong convergent and discriminant validity. Measurement invariance analyses demonstrated full invariance across grade levels and metric invariance across genders. Gender comparisons indicated more positive attitudes among males in mathematics, physics, engineering, and science and technology, whereas no gender differences emerged in chemistry. Correlational and regression analyses identified Mastery Orientation and Problem-Solving Confidence as positively related to attitudes across several STEM domains. Learned Helplessness and Emotional Control showed more discipline-specific associations, particularly for Mathematics and Engineering. The SDAS fills an important measurement gap by providing a reliable tool for assessing discipline-specific STEM attitudes and their psychological correlates, thereby supporting both research and educational interventions.

## 1. Introduction

Recently, increasing attention has been devoted to students’ engagement with science, technology, engineering, and mathematics (STEM) disciplines, given their central role in fostering innovation, economic development, and societal well-being (Bybee, 2013; OECD, 2019). STEM disciplines encompass a broad set of interconnected fields grounded in scientific inquiry, technological advancement, quantitative reasoning, and problem-solving, all of which are essential for addressing complex global challenges, such as climate change, public health, and digital transformation (McDonald, 2016; Tan et al., 2022). STEM is defined as an umbrella term encompassing science, technology, engineering, and mathematics as interrelated but distinguishable disciplinary domains (Bybee, 2013). However, its definition remains debated in the literature, ranging from an integrated, transdisciplinary curriculum to a set of separate school subjects (Staus et al., 2020). Consistent with the discipline-specific aim of the present instrument, we adopt the latter, disciplinary conceptualization: ‘STEM attitudes’ in this study refers specifically to students’ attitudes toward the STEM school disciplines as subjects of study, rather than to STEM-related careers, leisure activities, or informal learning experiences more broadly. This disciplinary approach was adopted for two main reasons: first, it aligns with the structure of Italian secondary education, where STEM content is taught through separate curricular subjects rather than an integrated STEM curriculum, making a discipline-specific measure more ecologically valid for capturing students’ school-based experiences; second, as detailed above, discipline-specific measurements are necessary to detect differential patterns of attitudes. Beyond their economic relevance, these disciplines also play a crucial role in equipping individuals with critical thinking skills, analytical competencies, and the ability to navigate an increasingly complex and technology-driven world (Hafni et al., 2020; Singh, 2024). In this sense, promoting students’ positive attitudes toward STEM is particularly important, as attitudes represent a key motivational resource that sustains engagement, supports learning processes, and influences the likelihood of pursuing further education and careers in these domains (Aduo et al., 2025; Murphy et al., 2019; Vennix et al., 2018). Students’ attitudes toward STEM encompass cognitive, affective, and behavioral components. The cognitive component reflects beliefs about one’s competence, performance, and the perceived difficulty or utility of a given discipline; the affective component captures enjoyment, curiosity, and emotional responses to the subject matter; and the behavioral component reflects intentions and tendencies to engage with the discipline, including career-related aspirations (Aduo et al., 2025; Demir et al., 2021; Kennedy et al., 2020; Murphy et al., 2019). However, despite the recognized importance of STEM fields, many students progressively disengage from these disciplines, with declines in participation often attributed to reduced interest, perceived difficulty, and lack of self-efficacy (Lytle & Shin, 2020; Maltese et al., 2014). Within this framework, subjects known as “hard sciences,” such as physics and chemistry, appear particularly vulnerable to motivational drop-offs. This happens because they are frequently perceived as abstract, cognitively demanding, and less intrinsically appealing than other domains of science education (Childs & Sheehan, 2009; Tytler & Osborne, 2012). Students frequently report physics and chemistry as being more difficult and less accessible than other STEM disciplines, leading to lower interest, reduced self-efficacy, and greater anxiety despite maintaining positive attitudes toward STEM more broadly (Hong et al., 2017; Sithole et al., 2017; Wong et al., 2023). Moreover, while general STEM attitudes are often influenced by broad beliefs about the societal relevance of science and technology, attitudes toward physics and chemistry are more strongly associated with classroom experiences, instructional practices, teacher support, perceived competence, and the intrinsic characteristics of the subjects (Mujtaba et al., 2018; Musengimana et al., 2021; Surya & Arty, 2021). Consequently, accurately assessing students’ attitudes toward STEM, particularly in physics and chemistry, requires psychometrically robust instruments capable of capturing their complexity across cognitive, affective, and motivational dimensions (Azevedo, 2015). Although several instruments have been developed to measure STEM-related attitudes and career interests (Guzey et al., 2014; Kier et al., 2014; Tyler-Wood et al., 2010; Unfried et al., 2015), existing measures often lack domain specificity or fail to adequately represent the nuanced psychological components underlying attitudes, particularly within secondary school populations.

Beyond domain-specific barriers, an accurate assessment of attitudes toward STEM must also account for crucial demographic variables that significantly shape adolescents’ academic pathways, particularly gender and grade. The international literature consistently highlights how female students tend to be underrepresented in “hard science” domains, a divergence largely driven by complex socialization processes, internalized cultural stereotypes, and lower perceived self-efficacy rather than innate cognitive differences (Bian et al., 2017; Cheryan et al., 2017; Su et al., 2009; M.-T. Wang & Degol, 2017). Similarly, research indicates that students’ attitudes toward STEM often fluctuate or progressively decline throughout their high school years, marking adolescence as a critical developmental period for academic and career decision-making (Lane et al., 2022; Potvin & Hasni, 2014; N. Wang et al., 2023).

Given the pervasiveness of these differences, it is methodologically imperative to ensure that the assessment tools used to measure these constructs are free of measurement bias across different subgroups. However, assessing these constructs remains challenging, particularly considering the heterogeneity of STEM disciplines. Among the few standardized instruments available, the most widely used is the Student Attitudes toward STEM (S-STEM) survey developed by Unfried et al. (2015), which assesses students’ attitudes toward science and technology, engineering, and mathematics. While the S-STEM provides a valuable multidimensional framework, it does not differentiate among specific scientific domains, nor does it capture discipline-specific motivational patterns that the literature has repeatedly emphasized, especially the lower levels of attitude and confidence typically associated with physics and chemistry compared to biology or other natural sciences (Osborne et al., 2003; Potvin & Hasni, 2014). In line with the parent S-STEM instrument (Unfried et al., 2015), the ‘Science and Technology’ factor retains the original, general science items of the S-STEM, which do not reference a specific scientific subdiscipline, while Physics and Chemistry were added as separate, discipline-specific extensions given their documented distinct motivational profile as ‘hard sciences.’ Therefore, ‘Science and Technology’ functions as a general/residual category paralleling the S-STEM’s original science items rather than as a subdiscipline coordinate with Physics and Chemistry.

To address this limitation, the present study builds upon the original S-STEM structure and introduces a newly developed instrument explicitly designed to assess students’ attitudes toward STEM disciplines, with a particular focus on physics and chemistry. To the best of our knowledge, no existing validated instrument specifically measures attitudes toward these “hard science” domains within the broader STEM context, despite their high relevance for educational trajectories and the well-documented motivational barriers associated with them (Childs & Sheehan, 2009; Tytler & Osborne, 2012).

Beyond attitudes alone, navigating the rigorous demands of these technical curricula requires a high degree of academic resilience and grit, as students must frequently manage the frustration of iterative failure and complex abstractions (Hall et al., 2022; Smith, 2021). Within this framework, problem-solving skills emerge as a critical competency, encompassing an individual’s confidence in their ability to resolve issues (problem-solving confidence), their proactive approach to challenges (problem-solving approach), and their emotional control during stressful tasks (emotional control) (Heppner & Petersen, 1982). High levels of problem-solving confidence and effective emotional regulation are positively associated with academic persistence in STEM (Afacan & Kaya, 2022), whereas an avoidant approach style often leads to disengagement (Tan et al., 2022). Closely linked to these skills are students’ motivational responses to obstacles, specifically the contrast between Mastery Orientation (MO), which reflects a focus on skill acquisition and a tendency to view obstacles as essential for the learning process, and Learned Helplessness (LH), characterized by the acquired belief that outcomes are independent of one’s efforts. While MO fosters a view of errors as opportunities for growth, thereby sustaining intrinsic motivation and autonomy (Dweck & Yeager, 2019), LH can act as a significant barrier, leading to passivity and the avoidance of subjects perceived as cognitively demanding, such as physics and chemistry (Miller & Srougi, 2021; Sorrenti et al., 2019). Consequently, mastery-oriented students are more likely to persist through the inherent difficulties of scientific inquiry, whereas those experiencing helplessness are at a higher risk of academic dropout.

Therefore, the current study aims to validate an adapted and extended version of the S-STEM, called the STEM Disciplines Attitude Scale (SDAS), for use with Italian high school students. Specifically, this study has five primary objectives: (1) to explore and confirm the factorial structure of the SDAS by comparing first-order and higher-order models; (2) to evaluate its internal consistency, as well as convergent and discriminant validity; (3) to test the measurement invariance (configural, metric, and scalar) of the instrument across gender (males vs. females) and grade levels (10th, 11th, and 12th grade); (4) to examine gender differences in students’ attitudes toward specific STEM domains; and (5) to examine the associations between problem-solving styles (specifically confidence, approach style, and emotional control), and motivational orientations (learned helplessness and mastery orientation) in relation to students’ attitudes across multidimensional STEM disciplines. This study addresses a significant methodological gap and provides researchers with a robust, fine-grained tool for examining attitudes toward the full spectrum of STEM subjects while elucidating the complex interplay between cognitive–behavioral factors and STEM attitudes.

## 2. Methods

### 2.1. Research Design

The present research adopted a sequential, three-study, cross-sectional design for scale development and validation, consistent with established guidelines for psychometric instrument construction (Boateng et al., 2018; DeVellis, 2016). Study 1 (*N* = 449) employed Exploratory Factor Analysis to identify the instrument’s underlying factor structure from an initial 30-item pool. Study 2 (*N* = 1858) used Confirmatory Factor Analysis to test and cross-validate this structure in an independent sample, together with measurement invariance testing across gender and grade level. Study 3 (*N* = 270) examined the nomological network of the instrument by testing associations with theoretically related constructs (problem-solving styles, learned helplessness, and mastery orientation) in a third independent sample. This staged approach—EFA, followed by independent CFA, followed by external validity testing—follows the recommended best practice for the development of psychological measurement instruments (Boateng et al., 2018).

### 2.2. Procedure

This study was conducted following the recommendations of the Ethical Code of the Italian Association of Psychology (AIP). All participants agreed to participate in the study and provided written informed consent in accordance with the Declaration of Helsinki (2013). For participants under 18 years of age, both legal tutors were fully informed about the study procedures and provided written consent on behalf of their children. Only students for whom signed informed consent forms were returned by themselves or by their tutors (in the case of minors) were allowed to participate in this study. Participants who did not provide consent were excluded.

The research protocol was approved by the Ethics Committee of the Centre for Research and Psychological Intervention (CeRIP) of the University of Messina (protocol number: 30465). Subsequently, the participants completed the questionnaires in one session. Data collection was conducted online using the LimeSurvey platform between December 2024 and May 2025 within the framework of the ConsapevolMENTE university orientation project promoted by the University of Messina. Across the overall project, 36 Sicilian high schools participated, located primarily in the city and province of Messina, with additional schools participating in the provinces of Siracusa and Ragusa. Different subsets of schools contributed to each of the three studies, which were based on fully independent samples. Study 1 involved a smaller subset of participating schools, Study 2 included the majority of schools, and Study 3 involved an additional independent subset. Schools voluntarily participated in the project, and the questionnaire was administered in collaboration with school principals and teachers during scheduled school activities. Student participation was entirely voluntary and individual based on personal interest; because data were not collected using a cluster-based design (e.g., intact classrooms), the dataset was treated as a non-nested structure with statistically independent observations. Also, although data were collected across multiple schools preliminary analyses indicated negligible clustering effects (ICC < 0.05), justifying the treatment of individual responses as statistically independent. Among students in the target grade range (10th–12th grade) who began the questionnaire, 2798 were eligible; of these, 2577 (92.1%) fully completed the survey and were retained for analysis, while 221 (7.9%) were excluded due to incomplete responses (participants exiting before reaching the final page) via listwise deletion. Because the online survey platform required each page to be completed before proceeding to the next, no item-level missingness occurred within completed questionnaires; consequently, all missingness in the present study is attributable to whole-questionnaire non-completion rather than to sporadic item non-response, making listwise deletion equivalent to retaining all cases with no missing data and not entailing the loss of otherwise-usable partial responses. The privacy and anonymity of all responses were strictly guaranteed, and participation required between 20 and 30 min.

## 3. Study 1—Exploratory Factor Analysis

### 3.1. Methods

#### 3.1.1. Participants

Study 1 involved 449 Italian 10th, 11th, and 12th grade students aged between 15 and 20 years (M = 16.94, SD = 1.01). The sample consisted of 240 female students (53.5%) and 205 male students (45.7%), and a small percentage (*N* = 4; 0.9%) preferred not to specify their gender. Most participants were Italian citizens (*N* = 436; 97.1%), with a smaller proportion being foreign students (*N* = 13; 2.9%). The students’ Socio-Economic Status (SES), determined by the Parent’s Highest Level of Education (PHLE), indicates a consistently high level of familial educational capital. Specifically, 44.1% (*N* = 198) were classified as having High SES (at least one parent holds a university degree), and 45.6% (*N* = 205) were classified as having Medium SES (highest family attainment is a high school diploma). Only 10.3% (*N* = 46) were categorized as having Low SES (neither parent attained a diploma).

#### 3.1.2. Instruments

A demographic questionnaire collected participants’ basic demographic information, including gender, age, nationality, parents or caregivers’ level of education, type of school attended (e.g., general or vocational school), and school grade level.

In Study 1, a questionnaire was developed based on the Students Attitudes toward STEM Survey (S-STEM)—Middle/High School version (Unfried et al., 2015). Of the 30 initial items, 22 were direct adaptations of existing S-STEM items: the Mathematics, Engineering, and Science and Technology subscales (six items each) were adapted with minimal wording changes from the corresponding S-STEM Math, Engineering and Technology, and Science subscales, respectively. Because the S-STEM does not include a Physics subscale, the six Physics items were newly developed by adapting the structure and wording of the S-STEM Mathematics items to the physics domain. The Chemistry subscale combined two newly developed items assessing perceived importance and curiosity, one item adapted from an otherwise unused S-STEM Mathematics item, and three items adapted from the S-STEM Engineering and Technology subscale. The original tool was translated into Italian using a forward-translation procedure, the wording was reviewed for cultural and educational appropriateness, and the items were adapted to the Italian high-school context. Content validity was formally assessed using Lynn’s (1986) Content Validity Index. Four experts in developmental and school psychology and psychometrics rated each of the 30 initial items for relevance on a 4-point scale (1 = not relevant, 4 = highly relevant). Item-level CVI (I-CVI) was computed as the proportion of experts rating each item 3 or 4; given the small panel size (*N* = 4 experts), the recommended acceptability threshold was I-CVI = 1.00 (Polit et al., 2007), rather than the ≥0.78 threshold used for larger panels (Lynn, 1986). All 30 items achieved an I-CVI of 1.00, yielding a Scale-CVI of 1.00 (both average and universal agreement) (Polit et al., 2007). No cognitive interviews or pilot testing with students were conducted prior to the main data collection process.

The first version of the developed questionnaire, called the STEM Disciplines Attitude Scale (SDAS), comprised 30 items, divided into five subscales: attitude toward mathematics (items 1–6), attitude toward physics (items 7–12), attitude toward chemistry (items 13–18), attitude toward engineering (items 19–24), and attitude toward science and technology (items 25–30). The items were formulated as statements with which students had to indicate their level of agreement on a five-point Likert scale (1 = strongly disagree; 5 = strongly agree). To mitigate response bias and control for social desirability, a subset of items was reverse-coded.

#### 3.1.3. Data Analysis

To identify the factorial structure underlying the adapted S-STEM questionnaire, the collected data were analyzed using Exploratory Factor Analysis (EFA) with R software (version 2026.07.1+147), using the lavaan (version 0.6-21) and semTools (version 0.5-8) packages. Prior to EFA, the adequacy of the sample and sphericity of the data were verified using the Kaiser–Meyer–Olkin (KMO) and Bartlett tests. These procedures ensured that the data were suitable for the factor analysis.

EFA was conducted on the entire set of items to explore the latent dimensions representing students’ attitudes toward STEM subjects.

Prior to conducting the EFA, item-level descriptive statistics were examined. Across the 30 initial items, skewness ranged from −0.811 to 0.461, and kurtosis ranged from −0.991 to 0.494 (*N* = 449), well within the conventional thresholds for approximate univariate normality (|skewness| < 2, |kurtosis| < 7; Curran et al., 1996), supporting the appropriateness of the maximum likelihood extraction method used.

Oblimin rotation was adopted because it is considered appropriate when factors are assumed to be correlated. The number of factors extracted was determined by combining statistical criteria, such as Parallel Analysis, and the theoretical interpretation of item loadings. Items were excluded from the final model if they failed to meet the primary loading threshold (>0.40) or exhibited substantial cross-loading, defined as a secondary loading ≥0.30, with a difference from the primary loading (Howard, 2016).

The model fit for the exploratory factor solution was evaluated using the Tucker–Lewis Index (TLI ≥ 0.90) and the Root Mean Square Error of Approximation (RMSEA ≤ 0.08), following Hu and Bentler’s (1999) conventional cutoff recommendations.

### 3.2. Results

The KMO measure yielded a value of 0.90, which is classified as excellent, indicating that the sample was highly adequate for the factor analysis. Bartlett’s Test of Sphericity was statistically significant (χ^2^(435) = 7277.55; *p* < 0.001), confirming that the correlation matrix was significantly different from an identity matrix, thus supporting the presence of an underlying common variance.

Parallel Analysis (Horn, 1965), based on 1000 random correlation matrices of equivalent dimensionality, indicated that five factors should be retained: the first five observed eigenvalues (9.075, 2.849, 2.614, 2.285, 1.478) exceeded both the mean (1.510, 1.442, 1.389, 1.344, 1.303) and the 95th percentile (1.578, 1.491, 1.431, 1.380, 1.338) of the corresponding simulated eigenvalues, whereas the sixth observed eigenvalue (1.104) did not exceed either simulated benchmark. This result converged with the theoretically expected five-factor structure and supported the retention of the five factors in the subsequent EFA.

The EFA supported a five-factor solution (Table 1), consistent with the theoretical framework of the S-STEM instrument (attitudes toward Mathematics, Physics, Chemistry, Engineering, and Science and Technology). These five factors collectively accounted for 53.80% of total variance. Factor correlations ranged from 0.054 (between Engineering and Chemistry) to 0.451 (between Science and Technology and Mathematics), empirically supporting the theoretical assumption of related dimensions and the use of oblique rotation.

Applying the pre-specified item-retention criteria described in Section 3.1.3 (primary loading threshold > 0.40; cross-loading threshold ≥ 0.30 with insufficient difference from the primary loading) to the initial 30-item, five-factor solution, which showed suboptimal fit (TLI = 0.871, RMSEA = 0.067; Table 1), identified five items failing to meet these criteria. This process resulted in a refined instrument comprising 25 items. Specifically, the removed items were: one Mathematics item (‘I am sure I could do advanced work in mathematics’), two Physics items (‘I would consider a career that uses physics’; ‘I am sure I could do advanced work in physics’), and two Chemistry items (‘I like to imagine designing and synthesizing new products/materials/molecules’; ‘In a chemist’s work, imagination and creativity are important’). All retained items displayed strong and clean factor saturations (≥0.45), and the final model demonstrated an excellent fit to the data, as evidenced by the Tucker–Lewis Index (TLI) of 0.926 and the Root Mean Square Error of Approximation (RMSEA) of 0.056. This five-factor, 25-item structure was then used in Study 2 for Confirmatory Factor Analysis.

## 4. Study 2—Confirmatory Factor Analysis and Measurement Invariance

### 4.1. Methods

#### 4.1.1. Participants

The sample utilized for Study 2 consisted of 1858 high school students from 10th, 11th, and 12th grades. The participants’ ages ranged from 15 to 20 years, with a mean age of 16.92 (SD = 0.96). The gender distribution showed a slight prevalence of female participants (*N* = 1019; 54.8%) compared to male participants (*N* = 820; 44.1%), with 1.0% (*N* = 19) identifying as other or not specifying their gender. The sample was highly homogeneous regarding nationality, with the vast majority being Italian nationals (*N* = 1793; 96.5%), and 65 students (3.5%) reporting foreign nationality. The students’ Socio-Economic Status (SES), determined by the Parent’s Highest Level of Education (PHLE), indicates a strong concentration in the high and medium educational tiers. Specifically, 39.6% (*N* = 735) were classified as having High SES (at least one parent holds a university degree), and 49.4% (*N* = 918) were classified as having Medium SES (highest family attainment is a high school diploma). Only 11.0% (*N* = 205) were categorized as having Low SES (neither parent attained a diploma).

#### 4.1.2. Instruments

A demographic questionnaire collected participants’ basic demographic information, including gender, age, nationality, parents or caregivers’ level of education, type of school attended (e.g., general or vocational school), and school grade level.

In accordance with the results of the EFA conducted in Study 1, the measurement model for the Confirmatory Factor Analysis (CFA) used the refined 25-item version of the SDAS. This final version consisted of five established related factors: Attitude toward Mathematics (5 items), Attitude toward Physics (4 items), Attitude toward Chemistry (4 items), Attitude toward Engineering (6 items), and Attitude toward Science and Technology (6 items). Responses for each item were collected using a 5-point Likert scale, where 1 indicates “strongly disagree” and 5 indicates “strongly agree”. To mitigate response bias and control for social desirability, a subset of items was reversed.

#### 4.1.3. Data Analysis

CFA was performed using the R statistical environment version (2026.07.1+147) (packages: lavaan version 0.6-21, semTools version 0.5-8). Multivariate normality was assessed on the final CFA item set using Mardia’s test, which indicated substantial departures from normality, supporting the use of a robust maximum likelihood estimator (MLR) appropriate for Likert-scale data.

To verify the overall fit of the model, the robust Comparative Fit Index (CFI) and Tucker–Lewis Index (TLI) (with values ≥ 0.90 as the threshold) were used as the primary indicators. Other key indices included the robust Root Mean Square Error of Approximation (RMSEA) and the Standardized Root Mean Square Residual (SRMR) (with a threshold value of 0.08).

To determine the optimal factorial structure, three alternative models were estimated and compared: a unidimensional model (M1), a first-order five-factor correlated model (M2), and a second-order model with a higher-order General STEM factor (M3). Nested model comparisons, including the evaluation of the optimized model with added residual covariances, were conducted using the scaled Satorra-Bentler Chi-square difference test (Δχ^2^) and information criteria, including the Akaike Information Criterion (AIC) and Bayesian Information Criterion (BIC), where lower values indicate a better fit.

To achieve an optimal fit, the initial model was refined by examining the Modification Indices (MIs). These indices suggest the opportunity to correlate the error residuals (residual covariances) between items that, despite belonging to distinct latent factors or sharing the same factor, could share a portion of specific variance (not explained by the constructs) owing to their thematic proximity or similar formulation (e.g., identical wording across different STEM fields). These correlations were added to the model sparingly and only when they were supported by theoretical justification.

Following the confirmation of structural validity, the psychometric properties of the five scales were assessed. Convergent validity was verified by examining the standardized factor loadings (λ), which had to be significant and ≥0.50, and by calculating the Average Variance Extracted (AVE), with a threshold value of ≥0.50. The Reliability of the scales was ascertained by calculating Cronbach’s alpha (α), McDonald’s omega (ω), and Composite Reliability (CR) coefficient, which was calculated from the CFA parameter estimates using the semTools package, with an acceptability criterion set at ≥0.70. Finally, Discriminant Validity was tested using the Heterotrait–Monotrait (HTMT) ratio of correlations, ensuring that all values were below the conservative threshold of 0.85. CFA was conducted on the full Study 2 sample (*N* = 1858). For gender-related analyses, including measurement invariance testing and comparisons between male and female students, the analytical sample comprised 1839 participants. Nineteen respondents (1.0%) reported a gender identity other than male or female or did not report their gender and were therefore not included in the analyses requiring binary gender grouping.

Therefore, Measurement Invariance across gender and grade levels was tested using a Multi-group CFA. Configural, metric, and scalar invariance were evaluated by comparing nested models using the change in CFI (ΔCFI ≤ 0.010) and RMSEA (ΔRMSEA ≤ 0.015) as the primary criteria.

Finally, to further explore gender-related differences in STEM attitudes, while appropriately accounting for measurement errors and differential item functioning, latent mean differences between male and female students were estimated directly within the partial scalar multi-group CFA model. Participants who selected “prefer not to specify” were excluded from these analyses, resulting in a final sample of *N* = 1839, with a mean age of 16.92 years (SD = 0.95). Effect sizes were estimated using Cohen’s d to quantify the magnitude of observed differences.

### 4.2. Results

Before evaluating the final psychometric properties, the fit of the three alternative measurement models was compared. The unidimensional model (M1) yielded a poor fit to the data (Robust CFI = 0.516, Robust TLI = 0.472, Robust RMSEA = 0.149). The second-order model (M3) showed acceptable fit indices (Robust CFI = 0.903, Robust TLI = 0.892, Robust RMSEA = 0.067); however, the scaled Satorra-Bentler chi-square difference test revealed that the first-order five-factor model (M2) provided a significantly better fit than M3 and had lower information criteria values (Table 2). Consequently, the first-order five-factor structure was retained as the baseline model for the CFA.

Following the addition of the theoretically justified residual covariances (specifically, between parallel items sharing identical wording, such as Mat4-Fis4 and Mat5-Fis5, and between semantically redundant items within the Engineering factor, Ing2-Ing5 and Ing3-Ing4), the optimized five-factor model demonstrated an overall good fit to the data (Scaled χ^2^(261) = 1684.65, *p* < 0.001), as indicated by the key fit indices: the robust CFI was 0.925, and the robust TLI was 0.914, both surpassing the recommended 0.90 threshold. Furthermore, the error terms were satisfactory, with a robust RMSEA of 0.060 (90% CI: 0.057–0.063), and an SRMR of 0.059. To address potential estimation biases stemming from the 5-point ordinal scale and observed multivariate non-normality, a sensitivity analysis was conducted on the final model using polychoric correlations and the WLSMV estimator. The ordinal model yielded acceptable fit indices (Robust CFI = 0.918, Robust TLI = 0.902, Robust RMSEA = 0.069, SRMR = 0.057) and standardized factor loadings highly consistent with the MLR estimation (ranging from 0.465 to 0.888).

Regarding Convergent Validity, all 25 items loaded significantly onto their respective factors (*p* < 0.001), with standardized factor loadings ranging from 0.507 to 0.867 for AVE (Table 3). Additionally, the Average Variance Extracted (AVE) was evaluated. Mathematics (AVE = 0.63), Physics (AVE = 0.62), and Science and Technology (AVE = 0.64) fully met the ≥0.50 criterion. Although the AVE values for Chemistry (AVE = 0.43) and Engineering (AVE = 0.41) were slightly below the strict threshold, their convergent validity was still considered adequate because their CR was well above the 0.70 threshold, according to Fornell and Larcker (1981). The internal consistency of the five subscales was acceptable to excellent, satisfying the ≥0.70 criterion for both Cronbach’s alpha (α = 0.727–0.909), McDonald’s omega (ω = 0.743–0.912), and CR (ranging from 0.74 to 0.91) (Table 3).

Finally, Discriminant Validity was confirmed, as the correlations between the latent factors were all statistically significant (*p* < 0.001) but ranged from moderate (r = 0.184 between Chemistry and Engineering) to strong (r = 0.579 between Engineering and Science and Technology), and the HTMT ratio criterion was met (maximum value = 0.548), indicating that each factor captures unique variance despite their meaningful interrelations (Table 4).

Measurement Invariance was tested across gender (males vs. females) and grade level (10th, 11th, and 12th grade) using Multi-Group CFA. For gender, the configural model showed a good fit (CFI = 0.910; RMSEA = 0.065). Metric invariance was fully supported (ΔCFI = −0.001, ΔRMSEA = −0.001), indicating that the factor loadings were equivalent across the groups. However, full scalar invariance was not perfectly achieved (ΔCFI = −0.013), with univariate score tests identifying 17 non-invariant intercepts (*p* < 0.01). To confirm the partial scalar invariance model, we iteratively released the equality constraints on the intercepts of the four items exhibiting the largest univariate score tests: Mat2, Ing2, Ing4, and Sci1. The resulting partial scalar model demonstrated a good fit (Robust CFI = 0.905, Robust RMSEA = 0.064) and met the criteria for invariance relative to the metric model (ΔCFI = −0.004, ΔRMSEA = 0.000). In particular, a minimum of four items remained fully invariant for each latent factor, well above the theoretical requirement of two invariant indicators per construct to establish partial scalar invariance (Putnick & Bornstein, 2016; Vandenberg & Lance, 2000). To assess the robustness of the partial invariance specification itself, we compared results obtained when releasing the top 2, top 4 (reported above), and top 6 non-invariant intercepts, ranked by univariate score-test statistics. Across all three specifications, model fit remained stable (Robust CFI: 0.903–0.907; Robust RMSEA: 0.064–0.065).

Regarding grade level, the configural model also demonstrated a good fit (CFI = 0.906; RMSEA = 0.067). Both metric (ΔCFI = −0.001, ΔRMSEA = −0.001) and full scalar invariance (ΔCFI = −0.004, ΔRMSEA = 0.000) were fully supported, as the changes in the fit indices were strictly within the recommended thresholds. These results confirm that the 25-item SDAS is fully scalar invariant across the high school triennium, allowing for reliable cross-sectional comparisons among classes and demonstrating metric equivalence across gender (Table 5).

Finally, gender differences in STEM attitudes were examined by comparing latent means within the established partial scalar invariance model. The results indicated statistically significant differences between male and female students in most domains, with males consistently exhibiting higher latent means. Specifically, significant latent differences emerged for Mathematics (Z = 4.16, *p* < 0.001, d = 0.21), Physics (Z = 5.17, *p* < 0.001, d = 0.26), Engineering (Z = 12.57, *p* < 0.001, d = 0.72), and Science and Technology (Z = 5.21, *p* < 0.001, d = 0.27). Conversely, no significant latent mean difference was found for Chemistry (Z = −1.03, *p* = 0.303).

## 5. Study 3—Correlational and Regression Analyses

### 5.1. Methods

#### 5.1.1. Participants

The sample utilized for Study 3 consisted of 270 10th, 11th, and 12th grade students. The participants’ age ranged from 15 to 20 years, with a mean age of 16.89 (SD = 0.98). Regarding gender, the sample was slightly predominantly female (*N* = 145; 53.7%) compared to male participants (*N* = 121; 44.8%), with a minor proportion (*N* = 4; 1.5%) identifying as other or not specifying their gender. Most of the student body were Italian nationals (*N* = 261; 96.7%), with 3.3% (*N* = 9) being foreign nationals. The students’ Socio-Economic Status (SES), determined by the Parent’s Highest Level of Education (PHLE), indicates a strong concentration in the high and medium educational tiers. Specifically, 34.4% (*N* = 93) were classified as having a High SES (at least one parent holds a university degree), and 48.5% (*N* = 131) were classified as having a Medium SES (highest family attainment is a high school diploma). Only 17.0% (*N* = 46) were categorized as having a Low SES (neither parent attained a diploma).

#### 5.1.2. Instruments

The individual perceptions of problem-solving styles were evaluated using the Problem-Solving Inventory (PSI; Heppner & Petersen, 1982), which consists of 26 items organized into three distinct subscales: problem-solving confidence (12 items), approach style (9 items), and emotional control (5 items). Responses to the PSI were collected on a 6-point Likert scale ranging from 1 (strongly disagree) to 6 (strongly agree), following the Italian validation established by Nota et al. (2009).

The constructs of Learned Helplessness (LH) and Mastery Orientation (MO) were assessed using the Learned Helplessness Questionnaire (LHQ; Sorrenti et al., 2015), a 13-item tool comprising 6 items for LH and 7 items for MO, where participants indicated their agreement on a 5-point Likert scale from 1 (not true) to 5 (absolutely true). This instrument has been extensively utilized in the Italian school psychology literature, as evidenced by the studies of Buzzai et al. (2021) and Filippello et al. (2018).

Finally, to quantify attitudes toward STEM disciplines, a 25-item SDAS questionnaire was administered (Appendix A), encompassing five specialized subscales for Mathematics (5 items), Physics (4 items), Chemistry (4 items), Engineering (6 items), and Science and Technology (6 items). Responses to the SDAS were provided on a standard 5-point Likert scale, ranging from 1 (strongly disagree) to 5 (strongly agree), with all reverse-scored items being appropriately recoded prior to statistical analysis to ensure data consistency.

#### 5.1.3. Data Analysis

As a preliminary step, descriptive and psychometric analyses were conducted on all variables included in Study 3. Specifically, means, standard deviations, skewness, and kurtosis were calculated to describe the distributional characteristics of the study variables. In addition, the internal consistency of the measures was assessed using Cronbach’s alpha, including the PSI, LHQ, and each of the five SDAS subscales.

To explore the relationships among the variables, Pearson’s correlation coefficients were calculated between problem-solving styles (confidence, approach style, and emotional control), motivational orientations (learned helplessness and mastery orientation), and the five specific domains of STEM attitude (Mathematics, Physics, Chemistry, Engineering, and Science and Technology).

Subsequently, a series of multiple linear regression analyses were performed to achieve a deeper understanding of how cognitive–behavioral and motivational factors are associated with interest in different scientific disciplines. This statistical approach makes it possible to assess the relationship between one or more independent variables and a dependent variable, estimating the unique contribution of each correlate to a comprehensive model. Unlike simple bivariate correlations, regression analysis identifies factors that maintain a significant role even when other variables are considered. Furthermore, this method provides an estimate of the total variance explained by the full model, offering a clear indication of the combined explanatory power of the examined psychological dimensions in relation to attitudes toward STEM.

### 5.2. Results

#### 5.2.1. Descriptive Statistics

Descriptive statistics were first examined for all variables included in Study 3. The means and standard deviations, along with the skewness and kurtosis indices, are reported in Table 6. Overall, the variables showed distributions that were approximately symmetric, with skewness and kurtosis values falling within the conventional thresholds generally considered indicative of acceptable departures from normality for subsequent regression analyses (|skewness| < 1 and |kurtosis| < 1.1; Kamath et al., 2025).

Internal consistency was subsequently assessed for all measures included in Study 3 using Cronbach’s alphas. Overall, the scales demonstrated acceptable to excellent internal consistency, with α coefficients ranging from 0.767 to 0.925 (Table 6). Most coefficients were above 0.80, indicating good reliability. Cronbach’s alpha for the Attitude toward Chemistry subscale (α = 0.767) was somewhat lower than that of the other SDAS subscales, although it remained above the commonly accepted threshold of 0.70 and was, therefore, considered acceptable.

#### 5.2.2. Correlations

The analysis revealed a complex pattern of association between psychological dimensions and interest in specific STEM domains. Pearson’s correlation coefficients (r) showed that Problem-Solving Confidence (PSC) had significant positive associations with attitudes toward all domains: Mathematics (r = 0.267, *p* < 0.001), Physics (r = 0.324, *p* < 0.001), Chemistry (r = 0.379, *p* < 0.001), Engineering (r = 0.321, *p* < 0.001), and Science and Technology (r = 0.290, *p* < 0.001).

Regarding the other dimensions of the Problem-Solving Inventory, Problem-Solving Approach (PSA) exhibited a more selective profile, correlating significantly with attitudes toward Chemistry (r = 0.211, *p* < 0.001) and Mathematics (r = 0.122, *p* < 0.05), while showing no significant relationship with Physics, Engineering, or Science and Technology. Similarly, Emotional Control (EC) displayed significant positive associations with attitudes toward Engineering (r = 0.175, *p* < 0.01), Mathematics (r = 0.167, *p* < 0.01), and Physics (r = 0.129, *p* < 0.05).

In terms of motivational orientations, Mastery Orientation (MO) proved to be a robust correlate for attitudes toward the STEM disciplines, with particularly strong links to Chemistry (r = 0.422, *p* < 0.001), Mathematics (r = 0.333, *p* < 0.001), Science and Technology (r = 0.273, *p* < 0.001), and Physics (r = 0.272, *p* < 0.001). Conversely, Learned Helplessness (LH) showed significant negative associations with attitudes toward Mathematics (r = −0.282, *p* < 0.001), Physics (r = −0.166, *p* < 0.01), and Chemistry (r = −0.153, *p* < 0.05). Notably, LH did not reach statistical significance in its association with Engineering or Science and Technology attitudes.

The relationships between problem-solving styles and motivational orientations provide further insight into the interplay among these constructs. Mastery Orientation (MO) was strongly and positively associated with Problem-Solving Confidence (r = 0.642, *p* < 0.001) and Problem-Solving Approach (r = 0.346, *p* < 0.001), while its relationship with Emotional Control remained non-significant. In contrast, Learned Helplessness (LH) exhibited a significant negative association with all dimensions of the PSI, most notably with Emotional Control (r = −0.607, *p* < 0.001), followed by Problem-Solving Approach (r = −0.421, *p* < 0.001), and Problem-Solving Confidence (r = −0.248, *p* < 0.001). The correlation matrix is presented in Table S1.

#### 5.2.3. Regression Analysis

A series of multiple linear regression analyses was conducted to investigate the unique associations of problem-solving styles (confidence, approach, and emotional control) and motivational orientations (Mastery Orientation and Learned Helplessness) with students’ attitudes across the five STEM domains (Table 7). In each model, the psychological constructs were entered as independent variables, while attitudes toward Mathematics, Physics, Chemistry, Engineering, and Science and Technology served as dependent variables.

First, the model examining attitude toward Mathematics was significant, [*F*(5, 264) = 10.74, *p* < 0.001]. Specifically, attitude was significantly and positively associated with Mastery Orientation [*t*(264) = 3.88, β = 0.299, *p* < 0.001] and negatively associated with Learned Helplessness [*t*(264) = −3.24, β = −0.236, *p* = 0.001].

Similarly, the regression model for Physics yielded significant results, [*F*(5, 264) = 7.93, *p* < 0.001]. Within this dimension, Problem-Solving Confidence emerged as the only significant positive correlate [*t*(264) = 2.90, β = 0.221, *p* = 0.004].

The analysis of attitude toward Chemistry also produced a significant model, [*F*(5, 264) = 13.67, *p* < 0.001]. In this case, both Mastery Orientation [*t*(264) = 3.60, β = 0.271, *p* < 0.001] and Problem-Solving Confidence [*t*(264) = 2.52, β = 0.184, *p* = 0.012] were found to be significantly and positively related.

Furthermore, the model for Engineering was significant, [*F*(5, 264) = 10.09, *p* < 0.001]. Attitude toward this domain was positively associated with Problem-Solving Confidence [*t*(264) = 3.94, β = 0.296, *p* < 0.001] and Emotional Control, [*t*(264) = 3.79, β = 0.284, *p* < 0.001], while Problem-Solving Approach showed a significant negative association [*t*(264) = −3.00, β = −0.207, *p* = 0.003].

Finally, the regression model focusing on attitude toward Science and Technology was significant, [*F*(5, 264) = 6.53, *p* < 0.001]. Both Mastery Orientation [*t*(264) = 2.35, β = 0.187, *p* = 0.019] and Problem-Solving Confidence [*t*(264) = 2.31, β = 0.178, *p* = 0.022] emerged as significant positive correlates for this discipline.

To assess the robustness of these findings, a supplementary set of analyses re-estimated all five regression models, including gender, grade level, and socioeconomic status as covariates (Table S2). The pattern of significant psychological predictors remained largely stable: Mastery Orientation and Problem-Solving Confidence retained their significant positive associations across most domains, and Learned Helplessness remained a significant negative correlate of Mathematics attitudes. For Engineering, both Problem-Solving Approach (B = −0.141, *p* = 0.025) and Emotional Control (B = 0.201, *p* = 0.001) remained significant predictors after adjustment, supporting the robustness of the suppression pattern discussed above. Female gender was independently and significantly associated with lower attitudes toward Mathematics, Physics, Engineering, and Science and Technology (all *p*s < 0.05), consistent with the gender differences, while socioeconomic status showed a significant positive association only with Physics and Science and Technology attitudes among students with high parental education. Grade level was not significantly associated with any of the domains. Notably, the Problem-Solving Approach showed a significant negative association with Attitude toward Engineering (β = −0.207, *p* = 0.003) despite a non-significant, near-zero bivariate correlation with this domain, a pattern consistent with statistical suppression (Paulhus et al., 2004). This effect proved robust in a supplementary analysis controlling for gender, grade, and socioeconomic status (B = −0.141, *p* = 0.025; Table S2), together with the positive effect of Emotional Control (B = 0.201, *p* = 0.001), supporting a genuine rather than an artifactual suppression pattern.

The diagnostic checks supported the validity of the regression models. Variance Inflation Factors were low and consistent across all five models (VIF range = 1.49–1.89; tolerance range = 0.53–0.67), indicating no problematic multicollinearity among predictors (Table 8). Cook’s distance values were small across all models (all M < 0.01, all individual values < 0.08), well below the conventional threshold of 1, indicating no unduly influential cases (Table 8). Shapiro–Wilk tests on model residuals indicated approximately normal distributions for the Mathematics (W = 0.995, *p* = 0.584), Chemistry (W = 0.994, *p* = 0.335), and Engineering (W = 0.995, *p* = 0.526) models; residuals for the Physics (W = 0.986, *p* = 0.009) and Science and Technology (W = 0.984, *p* = 0.005) models showed statistically significant, though minor, departures from normality—a common occurrence with this sample size given the sensitivity of the Shapiro–Wilk test, and not considered to substantially threaten the validity of the OLS estimates given the otherwise acceptable diagnostic profile (Table 8).

Breusch–Pagan tests indicated significant heteroscedasticity for the Engineering (LM = 13.04, df = 5, *p* = 0.023) and Science and Technology (LM = 12.13, df = 5, *p* = 0.033) models; no evidence of heteroscedasticity was found for Mathematics, Physics, or Chemistry (all *p*s > 0.09). To verify robustness, the Engineering and Science and Technology models were re-estimated using heteroscedasticity-consistent (HC3) standard errors. For Engineering, Problem-Solving Confidence (*p* < 0.001), Problem-Solving Approach (*p* = 0.006), and Emotional Control (*p* < 0.001) remained significant. For Science and Technology, Problem-Solving Confidence (*p* = 0.027) and Mastery Orientation (*p* = 0.027) remained significant (Table 8). The coefficient estimates were unchanged, and only the standard errors were adjusted. These results confirm that the pattern of findings reported in Table 7 is robust to the detected heteroscedasticity.

Given the number of coefficients tested across the five regression models (25 in total), a Benjamini–Hochberg false discovery rate (FDR) correction to guard against inflated Type I error from multiple comparisons has been applied. Eight of the ten coefficients significant at the uncorrected α = 0.05 level remained significant after FDR correction (Mathematics: Mastery Orientation, Learned Helplessness; Physics: Problem-Solving Confidence; Chemistry: Mastery Orientation, Problem-Solving Confidence; Engineering: Problem-Solving Confidence, Problem-Solving Approach, Emotional Control). The associations between Science and Technology attitude and both Mastery Orientation (*p* = 0.019) and Problem-Solving Confidence (*p* = 0.022) did not survive FDR correction and should be interpreted with caution.

## 6. Discussion

The present study aimed to address a key limitation in the STEM education literature: the lack of psychometrically robust instruments capable of distinguishing students’ attitudes toward specific disciplines, particularly in more abstract “hard science” domains, such as physics and chemistry. Although existing measures have provided valuable insights into general STEM attitudes, they frequently overlook the nuanced ways in which students gravitate toward specific disciplines, failing to distinguish what draws students to one subject over another (Mahmood et al., 2025). To overcome this limitation, the current study aimed to develop and validate the STEM Disciplines Attitude Scale (SDAS), a multidimensional instrument explicitly designed to assess students’ attitudes toward five distinct domains: science and technology, engineering, mathematics, physics, and chemistry.

The results of this study indicate that the primary goals of this study were successfully achieved. The refined 25-item version of the SDAS demonstrated strong psychometric properties, confirming its validity and reliability for use among Italian high school students.

### 6.1. Factorial Structure

Results from both exploratory (EFA) and confirmatory (CFA) analyses consistently converged on a five-factor solution, aligning with the theoretical distinction among the targeted STEM domains. In particular, the first-order five-factor model demonstrated a superior fit compared to both the unidimensional and second-order alternatives, indicating that students’ attitudes are more accurately represented as a set of correlated but distinct constructs rather than as a single overarching dimension or as a higher-order latent factor. This finding carries important theoretical implications. This supports the view that attitudes toward STEM disciplines are not a monolithic construct, but rather a multidimensional and domain-specific phenomenon (Jansen et al., 2019; Staus et al., 2020). From this perspective, students’ orientations toward different STEM disciplines may develop along partially independent trajectories, shaped by discipline-specific experiences, perceived difficulty, and contextual influences (Gajda, 2025; Maphosa et al., 2022; Regan & DeWitt, 2015; Sparks et al., 2019). Importantly, the clear differentiation between physics and chemistry as distinct factors empirically justifies their separation within the SDAS. By isolating these “hard science” domains, the scale captures subtleties that are typically obscured in more generalized instruments, thereby providing a more accurate representation of the specific motivational barriers and engagement patterns associated with these subjects (Corrigan et al., 2023; Coutinho, 2024; Glenn & Odeleye, 2024).

### 6.2. Reliability and Validity

With respect to the second study objective, the SDAS demonstrated good to excellent internal consistency across all five subscales in the samples examined (α range = 0.767–0.925), supporting the reliability of the discipline-specific attitude scores. The comparatively lower reliability observed for the Chemistry subscale is consistent with the more heterogeneous item origin of this subscale. Convergent and discriminant validity were further supported by the pattern of associations observed in Study 3: attitudes across all five STEM domains showed theoretically consistent positive associations with adaptive psychological resources, namely Mastery Orientation and Problem-Solving Confidence, and largely negative or null associations with Learned Helplessness, particularly within the “hard sciences.” Simultaneously, the differentiated strength and specificity of these associations across domains, rather than a uniform pattern common to all five subscales, provide evidence of discriminant validity, indicating that the SDAS subscales capture related but empirically distinguishable dimensions of STEM attitudes rather than a single undifferentiated construct.

### 6.3. Measurement Invariance

Further support for the robustness of the SDAS is provided by the results of the measurement invariance analyses across grade level and gender. Regarding grade level, full scalar invariance was established across students in the 10th, 11th, and 12th grades. This finding indicates that both the factor loadings and item intercepts are equivalent across cohorts, suggesting that students at different stages of high school interpret and respond to the items in a comparable manner. From a methodological standpoint, this is a particularly important result, as it supports the use of the SDAS for reliable comparisons across grade levels and makes the instrument especially well-suited for longitudinal research aimed at tracking developmental changes in STEM interest over time. A more nuanced pattern emerged for the gender invariance. While metric invariance was fully supported, indicating that the factor structure and the meaning of the latent constructs were equivalent for male and female students, scalar invariance was only partially achieved. This suggests that, although boys and girls possess similar attitudes toward STEM disciplines, they may differ slightly in their baseline levels of endorsement for certain items, particularly in domains such as engineering and science (Chan, 2022; Dunne, 2023; Msambwa et al., 2024; Onile-ere et al., 2021). This partial scalar invariance is theoretically meaningful and can be interpreted considering the extensive literature on gender stereotypes and socialization processes in STEM, which highlights how culturally embedded beliefs and differential experiences may shape students’ self-perceptions and responses, even when the underlying constructs are shared (Delaney & Devereux, 2019; Muenks et al., 2020; Plante et al., 2019; Su & Rounds, 2015; N. Wang et al., 2023).

### 6.4. Gender Differences in STEM Attitudes

Beyond measurement invariance, this study examined mean-level gender differences in attitudes toward STEM, providing further insight into domain-specific motivational patterns. The results revealed that male students reported significantly higher attitudes toward mathematics, physics, engineering, and science, whereas no significant gender differences emerged for chemistry.

These findings are consistent with a large body of literature documenting gender disparities in STEM, particularly in mathematically intensive and engineering-related fields (Chan, 2022; Cheryan et al., 2017, 2025; Cimpian et al., 2020; El-Hout et al., 2021; Su & Rounds, 2015). The especially large effect observed for engineering aligns with research highlighting this domain as one of the most gender-segregated within STEM (Bairoh, 2023; Blosser, 2017; Docherty et al., 2020; Ferreira et al., 2025; Gutfleisch & Nennstiel, 2025; Smith & Evans, 2024). Interestingly, the absence of significant differences in chemistry suggests that not all “hard sciences” are equally affected by gender disparities (Corrigan et al., 2023; Harsh et al., 2012; Montes et al., 2022; Rüschenpöhler & Markic, 2020). This supports the importance of adopting a domain-specific perspective when examining STEM motivation, as different disciplines may be shaped by distinct socialization processes, stereotypes, and educational experiences.

### 6.5. Psychological Correlates of STEM Attitudes

The present findings also provide significant insights into the psychological mechanisms associated with attitudes toward STEM, highlighting the roles of problem-solving styles and motivational orientations. Correlation analysis revealed that Problem-Solving Confidence and Mastery Orientation (MO) are consistently and positively linked across the entire STEM spectrum. This suggests that students who perceive themselves as capable of managing complex tasks and view academic challenges as opportunities for growth rather than insurmountable threats tend to cultivate a more positive attitude toward technical and scientific disciplines (Geertsema & Bolander Laksov, 2019; Valenzuela-Peñuñuri et al., 2024; Zhang & He, 2025). Conversely, Learned Helplessness (LH) demonstrated a significant negative association, particularly with the “hard sciences.” This confirms that a perceived lack of control over academic outcomes acts as a substantial psychological barrier, specifically in subjects characterized by high levels of abstraction and cognitive demand (Miller & Srougi, 2021; Sorrenti et al., 2019).

The multivariate results from the regression models offer a more nuanced understanding by identifying the unique contributions of these psychological factors to specific domains. Notably, Mastery Orientation and Learned Helplessness emerged as the primary correlates of attitude toward Mathematics, suggesting that interest in this field is deeply rooted in students’ motivational mindset and resilience to failure (Liu, 2021; Ozturk et al., 2024). In contrast, attitudes toward Physics and Chemistry were uniquely associated with Problem-Solving Confidence, consistent with the idea that self-efficacy beliefs are closely associated with more positive attitudes toward these cognitively rigorous subjects (Halim et al., 2018; Salame et al., 2026; Wilczek et al., 2022). A particularly distinctive pattern was observed for Engineering, where attitude toward this discipline was uniquely linked to high Emotional Control and a lower propensity for the Problem-Solving Approach style. This suggests that more positive attitudes toward engineering may be associated with a specific regulatory profile characterized by the ability to maintain emotional stability during complex, multi-stage technical challenges (Botejara-Antúnez et al., 2022; Ergin et al., 2020). Finally, the consistent association between Mastery Orientation with Science and Technology underscores the role of an intrinsic growth mindset in sustaining engagement and motivation with technological innovation (Hong et al., 2023; Hsieh & Yu, 2023; Subaşı, 2020). Collectively, these results indicate that while certain psychological resources are globally beneficial for attitudes toward STEM, each discipline appears to be associated with a specific configuration of cognitive–behavioral and motivational assets.

Taken together, these findings highlight the value of adopting a domain-specific perspective when examining attitudes toward STEM and provide support for the SDAS as a reliable and theoretically grounded instrument for advancing research and practice in STEM education, also highlighting its potential utility in identifying specific areas where gender-sensitive interventions may be most needed.

## 7. Conclusions

In conclusion, the present study contributes to the STEM education literature by providing a psychometrically sound, domain-specific instrument capable of capturing the multifaceted nature of students’ attitudes toward distinct STEM disciplines. By explicitly differentiating between general STEM domains and more abstract “hard sciences” such as physics and chemistry, the SDAS addresses a critical measurement gap and offers a more nuanced understanding of students’ motivational profiles in late adolescence.

The validated 25-item version of the scale demonstrated strong reliability, solid evidence of construct validity, and satisfactory measurement invariance across key demographic variables, supporting its applicability in both research and educational contexts. Thus, the SDAS represents a valuable tool for identifying discipline-specific patterns of engagement and disengagement in cross-sectional research. Its suitability for monitoring changes over time will require future studies to establish its test–retest reliability and responsiveness to change.

Beyond its psychometric properties, the current research elucidates the complex interplay between cognitive–behavioral and motivational factors in shaping attitudes toward sciences. The findings revealed that while Mastery Orientation and Problem-Solving Confidence were positively associated with STEM attitudes across most domains, disciplines such as Engineering and Mathematics showed additional discipline-specific associations with self-regulatory and emotional control variables. Furthermore, the consistent negative association between Learned Helplessness and attitudes toward the “hard sciences” identifies a potentially relevant factor for future educational interventions.

In addition to supporting the psychometric robustness of the SDAS, the present findings provide meaningful insights into gender-related patterns of attitudes toward STEM. The observed differences, with males reporting higher levels of attitude in several domains, further highlight the importance of addressing persistent gender disparities in STEM education.

Overall, these results underline the potential of the SDAS not only as a measurement tool but also as a practical resource for identifying targeted areas for intervention aimed at promoting balanced engagement in STEM fields.

### Limitations and Future Directions

Despite the strengths of the present study, some limitations should be acknowledged. First, the cross-sectional nature of the data prevents any causal inferences and limits the possibility of examining the developmental trajectories of attitudes toward STEM disciplines over time. Longitudinal studies would be necessary to better understand how students’ attitudes evolve throughout adolescence and to establish temporal relationships among the constructs assessed.

Second, the exclusive reliance on self-report measures may introduce potential biases, such as social desirability or response styles, which could affect the accuracy of reported attitude levels. Future studies could benefit from integrating multiple data sources, including behavioral indicators or teacher reports, to provide a more comprehensive assessment of students’ motivation in STEM domains.

Third, the residual covariances in the Confirmatory Factor Analysis were specified after inspecting the modification indices within the full Study 2 sample. Although grounded in robust semantic justifications (wording effects), this optimization was partially data-driven. Future studies should aim to cross-validate this modified structure using fully independent samples.

Fourth, the current analyses establish convergent validity exclusively from an internal perspective by utilizing factor loadings and composite reliability estimates. The study did not assess external convergent validity against an established concurrent measure of STEM attitudes. Future validation efforts should incorporate established criterion measures to further solidify the external nomological network of the scale.

Fifth, while the instrument development process included a formal expert review of content validity, it did not incorporate cognitive interviews or pilot testing with students prior to data collection, and the translation procedure relied solely on forward translation, without an independent back-translation and reconciliation step. This approach is consistent with the common practice for instruments that adapt and extend an already validated measure (S-STEM; Unfried et al., 2015) rather than developing an entirely new one. Nonetheless, future refinements of the SDAS, particularly the newly developed Physics items and the Chemistry items adapted from the Engineering subscale, would benefit from formal back-translation and cognitive interviews with students to further verify linguistic and conceptual equivalence. Additionally, the SDAS does not include Biology or Geology as separate domains. Unlike physics and chemistry, these disciplines are not typically perceived as equally abstract or mathematically demanding, and the existing literature does not document the same steep motivational decline that led to the development of the present instrument. We acknowledge this as a boundary of the scale’s current scope and note that extending the SDAS to Biology and Geology represents a valuable direction for future research. Additionally, the expert panel used to establish content validity was relatively small (*N* = 4). Although we applied the more conservative I-CVI threshold recommended for small panels (Polit et al., 2007), a larger and more diverse panel, including, for instance, STEM disciplinary experts alongside psychometricians, would strengthen future content-validity assessments of the SDAS.

Finally, the sample was geographically limited to Italian high school students, primarily from a specific regional context (Sicily). Although this provides important initial validation evidence, it may restrict the generalizability of the findings to other cultural and educational settings. Therefore, further research is needed to replicate and extend these results using more diverse and international samples.

Nonetheless, the findings of this study have several important practical implications and open promising directions for future research. From an applied perspective, the SDAS provides researchers and educators with a fine-grained tool to identify students’ attitude levels toward specific STEM domains.

Moreover, the domain-specific nature of the instrument suggests its potential utility in evaluating educational programs and interventions designed to foster STEM engagement. In particular, the SDAS could be employed in future pre-post or longitudinal designs to assess initiatives aimed at reducing gender disparities in STEM fields, allowing researchers to monitor whether such interventions differentially impact boys’ and girls’ attitudes toward specific disciplines.

Building on the current findings, future research should employ Structural Equation Modeling (SEM) to further investigate the complex pathways through which cognitive–behavioral and motivational factors interact. By moving beyond regression-based approaches, SEM would allow for the simultaneous estimation of latent constructs and the testing of mediated or moderated paths. Such advanced modeling could provide a more robust theoretical framework for understanding the internal mechanisms that sustain or hinder engagement and motivation in STEM pathways.

Future studies could strengthen the external validity of the SDAS by co-administering an established, non-domain-specific STEM attitude measure or discipline-specific instruments, allowing for a direct test of convergent and criterion validity against independent measures.

Future research may further leverage the SDAS to explore the contextual and psychological factors underlying discipline-specific attitudes, as well as to assess the long-term impact of targeted interventions in promoting more inclusive and sustained engagement in STEM education.

## Figures and Tables

**Table 1:** Factor Loadings and Communality (h^2^) Values for Exploratory Factor Analysis (*N* = 449). Factor loadings in bold indicate the highest saturation of an item on its designated factor.

Item	1. Science and Technology	2. Mathematics	3. Engineering	4. Physics	5. Chemistry	h^2^
Sci5	**0.871**					0.764
Sci4	**0.868**					0.706
Sci2	**0.748**					0.673
Sci6	**0.719**					0.611
Sci3	**0.676**					0.618
Sci1	**0.670**					0.625
Mat3		**0.862**				0.730
Mat1		**0.860**				0.728
Mat4		**0.810**				0.691
Mat5		**0.769**				0.585
Mat2		**0.542**				0.511
Mat6		0.359				0.708
Ing5			**0.735**			0.510
Ing2			**0.712**			0.563
Ing4			**0.664**			0.505
Ing6			**0.629**			0.600
Ing1			**0.579**			0.412
Ing3			**0.522**			0.291
Fis3				**0.888**		0.772
Fis1				**0.782**		0.609
Fis5				**0.773**		0.614
Fis4				**0.458**		0.391
Fis6				0.430		0.608
Fis2				0.381		0.629
Chi1					**0.692**	0.584
Chi3					**0.591**	0.480
Chi2					**0.583**	0.453
Chi5					**0.537**	0.452
Chi4	−0.311				−0.447	0.591
Chi6					0.364	0.802
**Eigenvalues**	3.79	3.07	2.57	2.25	1.76	
**Expl. Variance (%)**	15.2	12.3	10.3	9.0	7.0	

Maximum likelihood extraction, oblimin rotation. Model fit: TLI = 0.871, RMSEA = 0.067 [90% CI: 0.062, 0.072]. Bold = primary loading ≥ 0.40 on the designated factor. Fit indices reported here refer to the initial 30-item, five-factor solution, prior to the removal of the five items; the refined 25-item solution yielded improved fit (TLI = 0.926, RMSEA = 0.056), consistent with the pre-specified exclusion criteria (primary loading > 0.40; cross-loading ≥ 0.30). Items Mat6, Fis2, Fis6, Chi4, Chi6 are those subsequently removed. Chi4 showed a problematic negative cross-loading on Factor 1 (−0.311) alongside a below-threshold, negative primary loading (−0.447) on its own factor, providing clear empirical grounds for its exclusion beyond the general below-threshold criterion. A review of Chi4’s item content (‘I like to imagine designing and synthesizing new products/materials/molecules’) suggests a plausible substantive explanation for this pattern: unlike the other retained Chemistry items, which refer to chemistry as a school subject, this item’s emphasis on designing and synthesizing new products evokes an applied, technology-oriented framing conceptually closer to the Science-and-Technology factor—consistent with its negative cross-loading specifically on that factor. Fit indices reported here refer to the initial 30-item, five-factor solution, prior to the removal of the five items described in the text; the refined 25-item solution yielded improved fit (TLI = 0.926, RMSEA = 0.056). As a robustness check, an EFA using polychoric correlations was performed on the same Study 1 sample (*N* = 449), appropriate for ordinal Likert-type data. Principal axis factoring on the polychoric correlation matrix yielded a highly consistent five-factor structure (mean communality = 0.60). Critically, the same five items identified as problematic in the ML-based EFA (Mat6, Fis2, Fis6, Chi4, Chi6) showed weak primary loadings (range: 0.40–0.52) and/or substantial cross-loadings under the ordinal approach as well; notably, Chi4 again showed a negative loading on its own factor (−0.50) alongside a negative cross-loading on the Science-and-Technology factor (−0.45), replicating the pattern observed with ML estimation. This convergence across estimation methods supports the robustness of our item-retention decisions.

**Table 2:** Fit Indices for the Competing Measurement Models (*N* = 1858).

Model	Scaled χ^2^	df	CFI	TLI	RMSEA	SRMR	AIC	BIC
M1: Unidimensional	9251.69 *	275	0.516	0.472	0.149	0.125	129,652.2	129,928.1
M3: Second-Order	2115.34 *	270	0.903	0.892	0.067	0.068	120,661.2	120,964.7
**M2: First-Order**	2003.74 *	**265**	**0.908**	**0.896**	**0.066**	**0.061**	**120** **,** **537.5**	**120** **,** **868.6**

* *p* < 0.001. The model in bold (M2) represents the final retained model based on overall superior goodness-of-fit statistics.

**Table 3:** Unstandardized and Standardized Factor Loadings and Reliability/Validity Metrics for the Optimized 25-Item Model.

Factor	Item	B	SE	λ
***Attitude toward*** ***Mathematics*** AVE = 0.63 α = 0.89 ω = 0.89 CR = 0.89	Mat3	0.959	0.020	0.867 ***
	Mat1	1.000	—	0.846 ***
	Mat4	0.889	0.022	0.824 ***
	Mat5	0.909	0.023	0.776 ***
	Mat2	0.707	0.024	0.632 ***
***Attitude toward Physics*** AVE = 0.62 α = 0.86 ω = 0.87 CR = 0.87	Fis3	0.934	0.024	0.828 ***
	Fis1	1.000	—	0.821 ***
	Fis5	0.888	0.026	0.784 ***
	Fis4	0.766	0.030	0.700 ***
***Attitude toward*** ***Chemistry*** AVE = 0.43 α = 0.73 ω = 0.74 CR = 0.74	Chi3	1.135	0.040	0.749 ***
	Chi1	1.000	—	0.727 ***
	Chi2	0.661	0.042	0.546 ***
	Chi5	0.624	0.040	0.507 ***
***Attitude toward*** ***Engineering*** AVE = 0.41 α = 0.81 ω = 0.81 CR = 0.75	Ing6	1.271	0.062	0.745 ***
	Ing4	1.200	0.061	0.653 ***
	Ing1	1.000	—	0.633 ***
	Ing2	0.866	0.039	0.597 ***
	Ing5	0.837	0.045	0.569 ***
	Ing3	0.869	0.057	0.510 ***
***Attitude toward*** ***Science and Technology*** AVE = 0.64 α = 0.91 ω = 0.91 CR = 0.91	Sci4	1.158	0.025	0.859 ***
	Sci5	1.135	0.028	0.854 ***
	Sci1	1.000	—	0.785 ***
	Sci2	1.010	0.029	0.767 ***
	Sci3	0.928	0.024	0.767 ***
	Sci6	0.814	0.028	0.710 ***

B = Unstandardized factor loading; SE = Standard Error; λ = Standardized factor loading; AVE = Average Variance Extracted; α = Cronbach’s alpha; ω = McDonald’s omega. The unstandardized loading for the first item of each factor was fixed to 1.00 to set the metric of the latent variable; thus, no standard error was estimated for these marker items. *** *p* < 0.001.

**Table 4:** Discriminant Validity: Latent Factor Correlations and HTMT Ratios.

Factor	1	2	3	4	5
**1. Attitude toward Mathematics**	—	*0.435*	*0.286*	*0.291*	*0.475*
**2. Attitude toward Physics**	0.396	—	*0.343*	*0.297*	*0.391*
**3. Attitude toward Chemistry**	0.266	0.336	—	*0.173*	*0.548*
**4. Attitude toward Engineering**	0.326	0.303	0.184	—	*0.537*
**5. Attitude toward Science and Technology**	0.438	0.369	0.494	0.579	—

*Note.* The standardized latent factor correlations are presented below the diagonal. Heterotrait-Monotrait (HTMT) ratios are presented above the diagonal in italics. All latent correlations were statistically significant at *p* < 0.001. HTMT values < 0.85 indicate excellent discriminant validity.

**Table 5:** Measurement Invariance across Gender and Grade Levels.

Model	Scaled χ^2^	df	Robust CFI	Robust RMSEA	ΔCFI	ΔRMSEA	Invariance
**Gender (Males vs. Females)**							
***Configural***	2212.53	530	0.910	0.065	—	—	Yes
***Metric***	2253.76	550	0.909	0.064	−0.001	−0.001	Yes
***Scalar (Full)***	2522.00	570	0.896	0.067	−0.013	+0.003	No
***Scalar (Partial)***	2351.25	566	0.905	0.064	−0.004	0.000	Yes
**Grade Level (10th, 11th, 12th)**							
***Configural***	2616.73	795	0.906	0.067	—	—	Yes
***Metric***	2684.74	835	0.905	0.066	−0.001	−0.001	Yes
***Scalar***	2810.29	875	0.901	0.065	−0.004	−0.001	Yes

All χ^2^ values were statistically significant (*p* < 0.001). ΔCFI and ΔRMSEA represent the changes in fit indices compared to the previous less-constrained model. Strict criteria for invariance support are ΔCFI ≤ 0.010 and ΔRMSEA ≤ 0.015.

**Table 6:** Descriptive Statistics and Reliability Estimates for Study 3 Variables (*N* = 270).

*Variable*	*M*	*SD*	*Skewness*	*Kurtosis*	*α*
Problem-Solving Confidence	4.26	0.82	−0.51	1.08	0.880
Problem-Solving Approach	4.04	0.89	−0.04	−0.19	0.801
Emotional Control	3.33	1.03	0.26	−0.15	0.786
Learned Helplessness	2.72	0.99	0.24	−0.60	0.841
Mastery Orientation	3.51	0.79	−0.29	0.01	0.809
Attitude toward Mathematics	2.99	1.00	−0.07	−0.62	0.890
Attitude toward Physics	3.03	0.94	−0.18	−0.32	0.869
Attitude toward Chemistry	3.39	0.87	−0.11	−0.34	0.767
Attitude toward Engineering	3.12	0.83	0.16	0.06	0.837
Attitude toward Science and Technology	3.11	1.00	−0.06	−0.55	0.925

**Table 7:** Multiple Regression Analyses for Attitudes towards STEM Disciplines (*N* = 270).

DV	IV	B	SE	95% CI	β	t	*p*
**Attitude toward** **Mathematics** (*R*^2^ = *0.169*, *Adj. R*^2^ = *0.153*, *F*(*5*, *264*) = *10.74*, *p* < *0.001*)	PSC	0.041	0.091	[−0.14, 0.22]	0.033	0.45	0.652
	PSA	−0.120	0.077	[−0.27, 0.03]	−0.107	−1.56	0.120
	EC	0.039	0.073	[−0.11, 0.18]	0.040	0.53	0.593
	LH	−0.240	0.074	[−0.39, −0.09]	−0.236	−3.24	0.001
	MO	0.378	0.098	[0.19, 0.57]	0.299	3.88	<0.001
**Attitude toward** **Physics** (*R*^2^ = *0.131*, *Adj. R*^2^ = *0.114*, *F*(*5*, *264*) = *7.93*, *p* < *0.001*)	PSC	0.254	0.088	[0.08, 0.43]	0.221	2.90	0.004
	PSA	−0.130	0.074	[−0.28, 0.02]	−0.123	−1.77	0.078
	EC	0.078	0.070	[−0.06, 0.22]	0.085	1.13	0.261
	LH	−0.078	0.071	[−0.22, 0.06]	−0.082	−1.10	0.271
	MO	0.176	0.094	[−0.01, 0.36]	0.148	1.89	0.060
**Attitude toward** **Chemistry** (*R*^2^ = *0.206*, *Adj. R*^2^ = *0.191*, *F*(*5*, *264*) = *13.67*, *p* < *0.001*)	PSC	0.196	0.078	[0.04, 0.35]	0.184	2.52	0.012
	PSA	0.079	0.066	[−0.05, 0.21]	0.081	1.21	0.229
	EC	−0.049	0.062	[−0.17, 0.07]	−0.058	−0.79	0.429
	LH	−0.049	0.063	[−0.17, 0.08]	−0.055	−0.78	0.439
	MO	0.299	0.083	[0.14, 0.46]	0.271	3.60	<0.001
**Attitude toward** **Engineering** (*R*^2^ = *0.160*, *Adj. R*^2^ = *0.145*, *F*(*5*, *264*) = *10.09*, *p* < *0.001*)	PSC	0.299	0.076	[0.15, 0.45]	0.296	3.94	<0.001
	PSA	−0.192	0.064	[−0.32, −0.07]	−0.207	−3.00	0.003
	EC	0.228	0.060	[0.11, 0.35]	0.284	3.79	<0.001
	LH	0.097	0.061	[−0.02, 0.22]	0.116	1.57	0.117
	MO	0.085	0.081	[−0.07, 0.24]	0.081	1.05	0.295
**Attitude toward** **Science and Technology** (*R*^2^ = *0.110*, *Adj. R*^2^ = *0.093*, *F*(*5*, *264*) = *6.53*, *p* < *0.001*)	PSC	0.218	0.095	[0.03, 0.41]	0.178	2.31	0.022
	PSA	−0.142	0.080	[−0.30, 0.02]	−0.126	−1.78	0.076
	EC	−0.011	0.075	[−0.16, 0.14]	−0.011	−0.15	0.884
	LH	−0.083	0.077	[−0.24, 0.07]	−0.082	−1.08	0.280
	MO	0.237	0.101	[0.04, 0.44]	0.187	2.35	0.019

DV = Dependent Variable; IV = Independent Variable; B = unstandardized regression coefficient; SE = standard error; CI = confidence interval; β = standardized regression coefficient. IVs: PSC = Problem-Solving Confidence; PSA = Problem-Solving Approach; EC = Emotional Control; LH = Learned Helplessness; MO = Mastery Orientation.

**Table 8:** Regression Diagnostics for the Five STEM Attitude Models (*N* = 270).

Model	VIF (PSC/PSA/EC/LH/MO)	Cook’s D (M/Max)	Shapiro-Wilk Residuals (W, *p*)	Breusch–Pagan (LM, *p*)	HC3 Robustness Check
**Mathematics**	1.767/1.488/1.765/1.692/1.889	0.002/0.066	0.995, 0.584	9.47, 0.092	Not required (no heteroscedasticity detected)
**Physics**	1.767/1.488/1.765/1.692/1.889	0.001/0.078	0.986, **0.009**	7.68, 0.175	Not required (no heteroscedasticity detected)
**Chemistry**	1.767/1.488/1.765/1.692/1.889	0.001/0.047	0.994, 0.335	3.58, 0.612	Not required (no heteroscedasticity detected)
**Engineering**	1.767/1.488/1.765/1.692/1.889	0.001/0.044	0.995, 0.526	13.04, **0.023**	PSC, PSA, EC remained significant (all *p* < 0.01) with HC3 SE
**Science and Technology**	1.767/1.488/1.765/1.692/1.889	0.001/0.042	0.984, **0.005**	12.13, **0.033**	PSC and MO remained significant (both *p* = 0.027) with HC3 SE

PSC = Problem-Solving Confidence; PSA = Problem-Solving Approach; EC = Emotional Control; LH = Learned Helplessness; MO = Mastery Orientation. The VIF is identical across the five models because the IVs set is constant. Bold *p*-values indicate significant departures from the tested assumption. Where Breusch–Pagan tests indicated significant heteroscedasticity, models were re-estimated with heteroscedasticity-consistent (HC3) standard errors as a robustness check.

## Data Availability

The data that support the findings of this study are available from the corresponding author upon reasonable request.

## Supplementary Materials

The following supporting information can be downloaded at https://www.mdpi.com/article/10.3390/ejihpe16090125/s1, Figure S1: Parallel Analysis for the 25-item SDAS/Study 1 sample (*N* = 449). The scree plot displays the observed eigenvalues from the actual data (blue solid line, triangles) against the mean eigenvalues generated from simulated random data (red dotted line) and resampled data (red dashed line), each based on 1,000 iterations. Factors are retained when the observed eigenvalue exceeds the corresponding simulated/resampled eigenvalue. The observed eigenvalues exceed both reference lines for the first five factors and fall below them from the sixth factor onward, supporting the retention of a five-factor solution; Table S1: Correlational analysis results; Table S2: Covariate-Adjusted Regression Models.

## Abbreviations

The following abbreviations are used in this manuscript:

SDAS	STEM Disciplines Attitude Scale
STEM	Science, technology, engineering, and mathematics
EFA	Exploratory Factor Analysis
CFA	Confirmatory Factor Analysis
AIP	Italian Association of Psychology (Associazione Italiana di Psicologia)
CeRIP	Centre for Research and Psychological Intervention
SES	Socio-Economic Status
PHLE	Parent’s Highest Level of Education
S-STEM	Student Attitudes toward STEM
KMO	Kaiser–Meyer–Olkin
ML	Maximum Likelihood
TLI	Tucker–Lewis Index
RMSEA	Root Mean Square Error of Approximation
MLR	Robust maximum likelihood estimator
CFI	Comparative Fit Index
SRMR	Standardized Root Mean Square Residual
AIC	Akaike Information Criterion
BIC	Bayesian Information Criterion
MIs	Modification Indices
AVE	Average Variance Extracted
HTMT	Heterotrait–Monotrait
MO	Mastery Orientation
LH	Learned Helplessness
PSI	Problem-Solving Inventory
PSC	Problem-Solving Confidence
PSA	Problem-Solving Approach
EC	Emotional Control
LHQ	Learned Helplessness Questionnaire
SEM	Structural Equation Modeling

## Appendix A. The STEM Disciplines Attitude Scale (SDAS)

**Item**	**Italian**	**English**
***Mat1***	La matematica è stata per me la materia peggiore. *	Mathematics has been my worst subject. *
***Mat2***	Mi piacerebbe considerare una carriera in cui si usi la matematica.	I would like to consider a career that involves the use of mathematics.
***Mat3***	La matematica per me è difficile. *	Mathematics is difficult for me. *
***Mat4***	Sono uno studente bravo in ambito matematico.	I am a good student in mathematics.
***Mat5***	Riesco a fare bene la maggior parte delle materie, ma non riesco a fare un buon lavoro in matematica. *	I can do well in most subjects, but I cannot do a good job in mathematics. *
***Fis1***	La fisica è stata per me la materia peggiore. *	Physics has been my worst subject. *
***Fis3***	La fisica per me è difficile. *	Physics is difficult for me. *
***Fis4***	Sono uno studente bravo in ambito fisico.	I am a good student in physics.
***Fis5***	Riesco a fare bene la maggior parte delle materie, ma non riesco a fare un buon lavoro in fisica. *	I can do well in most subjects, but I cannot do a good job in physics. *
***Chi1***	Non riesco a capire perché sia importante lo studio della chimica. *	I cannot understand why studying chemistry is important. *
***Chi2***	Lo studio della chimica suscita in me curiosità.	Studying chemistry sparks my curiosity.
***Chi3***	Posso ottenere buoni voti in chimica.	I can achieve good grades in chemistry.
***Chi5***	Studiando chimica potrei migliorare le cose che le persone usano ogni giorno.	By studying chemistry, I could improve the things people use every day.
***Ing1***	Sono curioso di sapere come lavora un ingegnere elettronico.	I am curious to know how an electronic engineer works.
***Ing2***	Studiando ingegneria potrei migliorare le cose che le persone usano ogni giorno.	By studying engineering, I could improve the things people use every day.
***Ing3***	Sono bravo a costruire e riparare oggetti.	I am good at building and repairing objects.
***Ing4***	Sono interessato ai meccanismi che permettono alle macchine di funzionare.	I am interested in the mechanisms that allow machines to function.
***Ing5***	L’ingegneria mi permetterà di inventare cose utili.	Engineering will allow me to invent useful things.
***Ing6***	Credo di poter avviare una carriera in ambito ingegneristico.	I believe I can pursue a career in the field of engineering.
***Sci1***	So di poter fare bene in campo scientifico/tecnologico.	I know I can do well in the field of science and technology.
***Sci2***	Gli studi scientifici saranno importanti nel mio futuro lavorativo.	Scientific studies will be important for my future career.
***Sci3***	Sono sicuro di poter fare progressi in ambito scientifico/tecnologico.	I am confident that I can make progress in the field of science and technology.
***Sci4***	Vorrei considerare una carriera in campo scientifico/tecnologico.	I would like to consider a career in the field of science and technology.
***Sci5***	Mi aspetto di usare le mie conoscenze scientifico/tecnologiche una volta finiti gli studi scolastici.	I expect to use my scientific and technological knowledge once I finish my studies.
***Sci6***	Le mie conoscenze scientifico/tecnologiche mi aiuteranno nella vita.	My scientific and technological knowledge will help me in life.
* Reverse-scored items.		
