# Supplementary material for: Psychometric Validation of the STEM Disciplines Attitude Scale (SDAS): Investigating Measurement Invariance and Psychological Correlates in Italian High School Students
Source: Eur J Investig Health Psychol Educ. 2026 Aug 26;16(9):125. doi: 10.3390/ejihpe16090125 (PMC13605343; doi:10.3390/ejihpe16090125)
Supplement: Supplementary file 1 [file ejihpe-16-00125-s001.zip › ejihpe-4466050-supplementary.pdf]

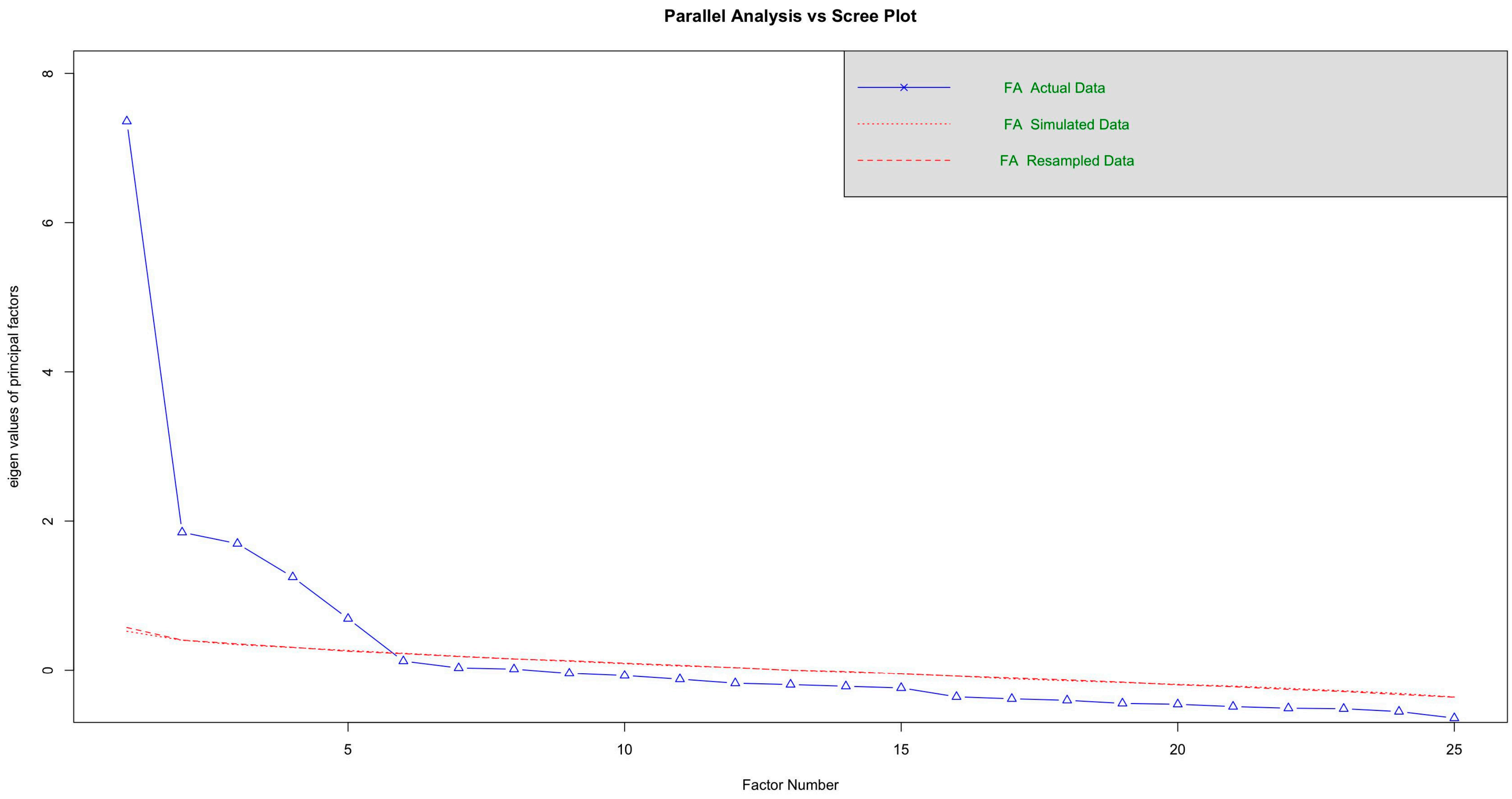

Figure S1. Parallel Analysis for the 25-item SDAS/Study 1 sample ($N = 449$). The scree plot displays the observed eigenvalues from the actual data (blue solid line, triangles) against the mean eigenvalues generated from simulated random data (red dotted line) and resampled data (red dashed line), each based on 1000 iterations. Factors are retained when the observed eigenvalue exceeds the corresponding simulated/resampled eigenvalue. The observed eigenvalues exceed both reference lines for the first five factors and fall below them from the sixth factor onward, supporting the retention of a five-factor solution.

Table S1. Correlational analysis results. (Note: * p < .05, ** p < .01, *** p < .001)

	1	2	3	4	5	6	7	8	9	10
**1. Problem Solving Confidence**	—									
**2. Problem Solving Approach**	0.218***	—								
**3. Emotional Control**	0.166**	0.463***	—							
**4. Learned Helplessness**	-0.248***	-0.421***	-0.607***	—						
**5. Mastery Orientation**	0.642***	0.346***	0.094	-0.196**	—					
**6. Attitude toward Mathematics**	0.267***	0.122*	0.167**	-0.282***	0.333***	—				
**7. Attitude toward Physics**	0.324***	0.050	0.129*	-0.166**	0.272***	0.414***	—			
**8. Attitude toward Chemistry**	0.379***	0.211***	0.069	-0.153*	0.422***	0.298***	0.302***	—		
**9. Attitude toward Engineering**	0.321***	-0.031	0.175**	-0.059	0.203***	0.297***	0.319***	0.218***	—	
**10. Attitude toward Science**	0.290***	0.007	0.027	-0.103	0.273***	0.510***	0.410***	0.544***	0.471***	—

Table S2. Covariate-Adjusted Regression Models ($N = 270$).

DV	IV	B	SE	95% CI	t	p
**Attitude toward Mathematics** ***($R^2 = .198$, Adj. $R^2 = .167$, $F(10,259) = 6.39$, $p < .001$)***	PSC	0.012	0.092	[−0.17, 0.19]	0.13	.896
	PSA	−0.106	0.078	[−0.26, 0.05]	−1.36	.176
	EC	0.036	0.073	[−0.11, 0.18]	0.49	.624
	LH	−0.209	0.074	[−0.36, −0.06]	−2.81	.005
	MO	0.400	0.098	[0.21, 0.59]	4.08	<.001
	Female	−0.277	0.118	[−0.51, −0.05]	−2.36	.019
	Other/unspec. gender	−0.668	0.470	[−1.59, 0.26]	−1.42	.157
	Grade	−0.000	0.070	[−0.14, 0.14]	−0.00	.998
	SES Medium	0.149	0.163	[−0.17, 0.47]	0.91	.363
	SES High	0.244	0.174	[−0.10, 0.59]	1.40	.161
**Attitude toward Physics** ***($R^2 = .185$, Adj. $R^2 = .154$, $F(10,259) = 5.90$, $p < .001$)***	PSC	0.207	0.087	[0.04, 0.38]	2.39	.018
	PSA	−0.131	0.074	[−0.28, 0.01]	−1.78	.077
	EC	0.090	0.069	[−0.05, 0.23]	1.30	.194
	LH	−0.038	0.070	[−0.18, 0.10]	−0.54	.591
	MO	0.203	0.093	[0.02, 0.39]	2.19	.029
	Female	−0.249	0.111	[−0.47, −0.03]	−2.24	.026
	Other/unspec. gender	−0.644	0.445	[−1.52, 0.23]	−1.45	.149
	Grade	−0.081	0.066	[−0.21, 0.05]	−1.23	.218
	SES Medium	0.166	0.154	[−0.14, 0.47]	1.08	.281
	SES High	0.438	0.164	[0.11, 0.76]	2.66	.008
**Attitude toward Chemistry** ***($R^2 = .211$, Adj. $R^2 = .181$, $F(10,259) = 6.94$, $p < .001$)***	PSC	0.200	0.079	[0.04, 0.36]	2.52	.012
	PSA	0.065	0.068	[−0.07, 0.20]	0.96	.340
	EC	−0.041	0.063	[−0.17, 0.08]	−0.65	.514
	LH	−0.047	0.064	[−0.17, 0.08]	−0.73	.468
	MO	0.282	0.085	[0.12, 0.45]	3.33	.001
	Female	0.084	0.102	[−0.12, 0.28]	0.82	.411
	Other/unspec. gender	−0.245	0.407	[−1.05, 0.56]	−0.60	.547
	Grade	0.043	0.060	[−0.08, 0.16]	0.71	.478
	SES Medium	0.076	0.141	[−0.20, 0.35]	0.54	.592
	SES High	0.106	0.151	[−0.19, 0.40]	0.71	.481
**Attitude toward Engineering** ***($R^2 = .255$, Adj. $R^2 = .227$, $F(10,259) = 8.89$, $p < .001$)***	PSC	0.265	0.073	[0.12, 0.41]	3.63	<.001
	PSA	−0.141	0.062	[−0.26, −0.02]	−2.26	.025
	EC	0.201	0.058	[0.09, 0.32]	3.47	.001
	LH	0.121	0.059	[0.00, 0.24]	2.04	.042
	MO	0.136	0.078	[−0.02, 0.29]	1.75	.082
	Female	−0.518	0.094	[−0.70, −0.33]	−5.52	<.001
	Other/unspec. gender	−0.560	0.374	[−1.30, 0.18]	−1.50	.136
	Grade	0.037	0.055	[−0.07, 0.15]	0.67	.502
	SES Medium	−0.036	0.130	[−0.29, 0.22]	−0.28	.782
	SES High	−0.037	0.138	[−0.31, 0.24]	−0.27	.789
**Attitude toward Science & Technology** ***($R^2 = .159$, Adj. $R^2 = .127$, $F(10,259) = 4.91$, $p < .001$)***	PSC	0.189	0.094	[0.00, 0.37]	2.01	.045

	PSA	−0.139	0.080	[−0.30, 0.02]	−1.73	.085
	EC	−0.009	0.075	[−0.16, 0.14]	−0.12	.902
	LH	−0.045	0.076	[−0.20, 0.11]	−0.59	.554
	MO	0.245	0.100	[0.05, 0.44]	2.44	.015
	Female	−0.259	0.121	[−0.50, −0.02]	−2.14	.033
	Other/unspec. gender	−1.118	0.482	[−2.07, −0.17]	−2.32	.021
	Grade	0.081	0.071	[−0.06, 0.22]	1.13	.260
	SES Medium	0.254	0.167	[−0.08, 0.58]	1.52	.129
	SES High	0.385	0.178	[0.03, 0.74]	2.16	.032

DV = Dependent Variable; IV = Independent Variable; B = unstandardized regression coefficient; SE = standard error; CI = confidence interval; β = standardized regression coefficient. IVs: PSC = Problem-Solving Confidence; PSA = Problem-Solving Approach; EC = Emotional Control; LH = Learned Helplessness; MO = Mastery Orientation; SES = Socio-Economic Status
